# Appropriateness of ambulatory antibiotic prescribing in South Carolina, 2012–2017

**DOI:** 10.1017/ash.2024.500

**Published:** 2025-01-15

**Authors:** Pamela Bailey, Majdi N Al-Hasan, Julie Royer, Max Habicht, Julie Ann Justo, P. Brandon Bookstaver, Sharon Weissman, Hana R Winders

**Affiliations:** 1 University of South Carolina School of Medicine, Columbia, SC, USA; 2 Department of Internal Medicine, Division of Infectious Diseases, Prisma Health Midlands, Columbia, SC, USA; 3 South Carolina Revenue and Fiscal Affairs Office, Columbia, SC, USA; 4 South Carolina Department of Health and Environmental Control; Columbia, SC, USA; 5 University of South Carolina College of Pharmacy; Columbia, SC, USA; 6 Department of Pharmacy, Prisma Health Midlands, Columbia, SC, USA

## Abstract

This population-based cohort study examines the appropriateness of antibiotic prescribing in South Carolina via aggregated pharmacy claims data matched with diagnosis codes from medical claims. Inappropriate antibiotic prescribing decreased from 30.2% in 2012 to 22.6% in 2017 (*P* < 0.001) and was more common in adults >40 years old.

## Introduction

Approximately 90% of antimicrobials are prescribed in the ambulatory setting, and in 2010-2011, per 1000 US population, 353 of 506 antibiotic prescriptions were considered inappropriate.^1,2^ Acute upper respiratory tract infections (ARTI) are the most common offenders: sinusitis was the single diagnosis associated with the most antibiotic prescriptions per 1000 population, followed by suppurative otitis media and pharyngitis.^2^ These ARTI led to 221 antibiotic prescriptions annually per 1000 population in the United States, but only 111 per 1000 population were estimated to be appropriate for these conditions.^2^

Prior work in South Carolina has examined temporal trends in ambulatory antibiotic prescription fill rates with examination of the influence of age, gender, and location.^3^ This population-based cohort study examines the appropriateness of antibiotic prescribing in South Carolina from 2012 through 2017, with particular focus on age and gender.

## Methods

**Figure 1.**
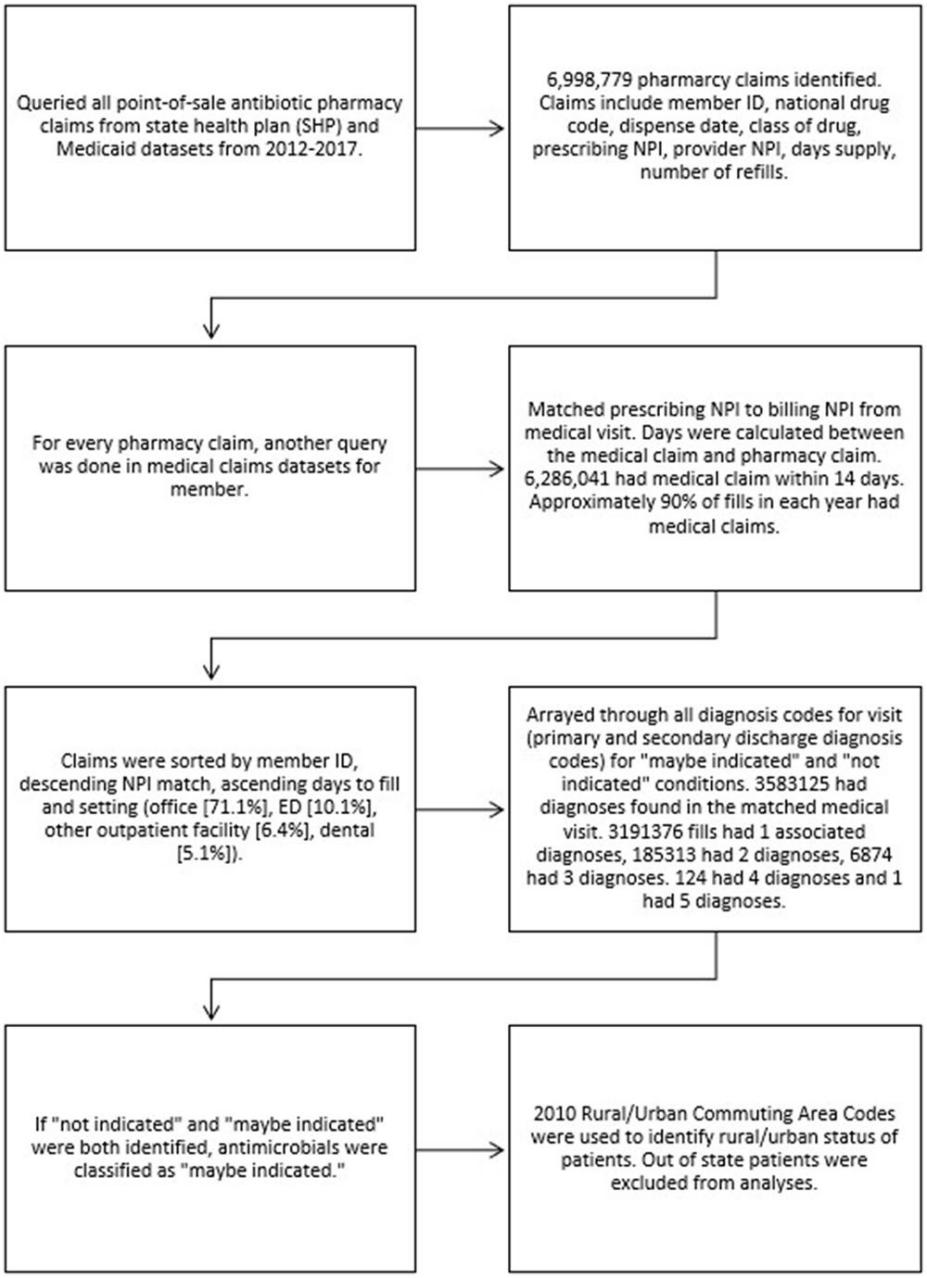
Methodology for creating dataset.

Through the South Carolina Revenue and Fiscals Affairs office, aggregated Medicaid and State Employee Health Plan pharmacy claims for ambulatory oral antibiotics were used to estimate community antibiotic prescription fill amounts, matched via national provider identifier (NPI) with International Classification of Diseases (ICD) diagnosis codes, either 9^th^ or 10^th^ revision from medical claims within 14 days of the pharmacy claim for individuals aged ≤64 years from January 1, 2012, to December 31, 2017 [Figure 1, supplement]. The ≥ 65-year-old cohort was excluded as not captured at high frequency in this dataset; regardless, these data represent nearly 30% of the South Carolina population. Data were then filtered to the ambulatory setting and patients from whom relevant demographic and diagnosis variables could be extrapolated. Appropriateness of antibiotics was defined as “maybe indicated” or “not indicated” based on ICD codes based on classification used in prior reports.^4^ All diagnoses codes from the matched visit were screened, and if diagnoses included both a “maybe indicated” and “not indicated,” the prescription was coded as “maybe indicated.” Chi-square or student t-tests were used as appropriate to examine overall temporal trend in appropriateness and the trends across age group and gender.

## Results

Overall, 6,286,041 antibiotic prescriptions were matched through medical and pharmacy claims data and national provider identifiers (NPI) linkage (89.8% of pharmacy claims connected to medical claim). When filtering to ambulatory setting and patients for whom data extrapolation was possible, 3,383,688 prescriptions were included in the analysis.

Overall, 26.8% of antibiotic prescriptions filled during the 6-year period were “not indicated” [Table 1]. This decreased from 2012 to 2017 from 30.2% to 22.6%, (*P* < 0.001). Each individual year also showed improved trend (Table 1).

**Table 1.** Demographic data and results

		Maybe Indicated [n, %]	Not Indicated [n, %]
By Year	2012	402955 (69.8)	174005 (30.2)
	2013	391559 (70.0)	168224 (30.0)
	2014	383717 (71.2)	155584 (28.8)
	2015	400299 (73.2)	146293 (26.8)
	2016	447522 (77.1)	133138 (22.9)
	2017	449198 (77.4)	131194 (22.6)
	total	2475250 (73.2)	908438 (26.8)
By Gender	Female	1451140 (72.9)	539419 (27.1)
	Male	1024110 (73.5)	369019 (26.5)
By Age Group	0–17yo	1694860 (76.2)	529942 (23.8)
	18–39yo	406163 (71.7)	160571 (28.3)
	40–64yo	374227 (63.2)	217925 (36.8)
By Geography	Rural	527408 (70.4)	221453 (29.6)
	Urban	1947842 (73.9)	686985 (26.1)

All p values <0.0001

The most common “maybe indicated” conditions were suppurative otitis media (n = 899663, 35.3%), sinusitis (n = 745585, 29.2%), and acute bacterial tonsillitis (n = 318798, 12.5%). The most common “not indicated” conditions were viral upper respiratory infections (n = 365483, 35.4%), bronchitis (n = 249810, 24.2%), and serous otitis media (n = 122145, 11.8%).

While females filled more antibiotic prescriptions (1,990,559 vs 1,393,129, *P* < 0.0001), they were slightly more likely than males to have a “not indicated” condition (27.1% vs 26.5%, *P* < 0.0001) (Table 1). Prescribing for “not indicated” conditions was significantly (*P* < 0.001) more likely in adults aged 40–64 (36.8%) than those aged 18–39 (23.8%), and <18 (23.8%) (Table 1).

## Discussion

The observed decline in inappropriate ambulatory antibiotic prescribing from 2012 to 2017 is welcome, though further stewardship efforts are needed particularly in males and adults ≥40 years old. The most common diagnoses for both “not indicated” and “maybe indicated” antibiotic prescriptions were ARTI.

A 2015 meta-analysis showed that women in the 16–54 age group receive significantly higher number of prescriptions of cephalosporins and macrolides in primary care than men do, presumed by the authors to be related to the higher prescriptions in respiratory tract infections as the fluoroquinolones prescribed for primarily urinary tract infections remained balanced between the genders.^5^ Women have more acute visits for respiratory infection than men, despite men having more risk factors for chronic respiratory conditions related to social behaviors (e.g. smokers, illicit drug user, regular and heavy alcohol use) which put them at risk for acute infections.^6,7^ While women received more antibiotic prescriptions in our data (3:2 ratio), there was more appropriate antibiotic prescribing for diagnoses in males; this should be further researched with considerations for the differences in health-seeking behaviors and comorbid conditions.

In the 2010 US Census data, 33.7% of the population of South Carolina resided in a rural area.^8^ Rural areas may be a particularly difficult area for antimicrobial stewardship. Rural visits, particularly in the southern United States, are a significant contributor to inappropriate antimicrobial prescriptions.^3^ In a 2019 survey, 10% of providers in rural areas had not heard of antimicrobial stewardship but were generally (84.3%) interested in learning more and acknowledged that antibiotics are overprescribed and inappropriate use leads to resistance.^9^ Providers who saw ≥50 patients a week felt more pressured to prescribe antibiotics, as well as if the visit were ≤20 minutes. Only 42.9% of prescribers selected that 90-98% rhinosinusitis are viral, with only 5.7% recommending supportive care without antibiotics; viral sinusitis was one of the most common “not indicated” diagnoses in this analysis.^9^

Behavioral and unmeasured factors, such as patient, prescriber, health care system, health care payer, and local/state policies, may impact prescribing rates. Additional factors such as the setting (e.g., urgent care or emergency department) or patient/provider relationship also likely play a significant role in prescribing patterns.^10^ In an analysis of patient, physician, and practice characteristics, it was noted that family medicine providers, female gender, and self-report race/ethnicity of white or Hispanic were significantly associated with inappropriate antibiotic prescribing around ARTI.^6^ They also noted that a group with low prescribers was likely to have overall low prescribers, whereas groups with high prescribers were 1.3 times more likely to have a second high prescriber.^6^ In a study where patient characteristics were similar, it is the providers who had significant variability in prescribing.^11^ Patients who saw high prescribers received 3.0 more fills per 100 people or 14.6% more fills over the subsequent year, compared with those seen by low prescribers.^11^ The patients with prior fills were also more likely to have repeat upper respiratory tract visits in the subsequent year, with a relative increase in prescribing across quartiles at these subsequent visits at 2.8%.^11^ Diagnoses were also different in high prescribers compared to low prescribers; the high prescribers tended to use diagnoses where antibiotics may be indicated compared to low prescribers using more viral conditions where antibiotics are not indicated.^11,12^

This study has several limitations. Extrapolating from coding and claims data is limited in assessing accuracy of the data, and the provider may have coded the diagnoses inaccurately.^12^ Additionally, prescribers may have intentionally coded inaccurately to avoid scrutiny of inappropriate prescribing. There may be limited ability to generalize these data to other states or geographic regions. The ≥ 65-year-old cohort was excluded from analysis due to inability to capture them in this data set (only 1.0% initial data), which limits generalizability into that population. There was also ongoing national and international work to increase awareness of antimicrobial stewardship during the period of this study, which may confound results. Ecologic fallacy is also a concern in using administrative data such as this.

While the reduction in inappropriate antibiotic prescribing is encouraging, there is still significant work to achieve the goals of antimicrobial stewardship in the ambulatory setting.

## Supporting information

Bailey et al. supplementary material Bailey et al. supplementary material

## Supplementary material

For supplementary material accompanying this paper visit http://doi.org/10.1017/ash.2024.500. click here to view supplementary material

## References

[1] Duffy E , Ritchie S , Metcalfe S , Van Bakel B , Thomas MG . Antibacterials dispensed in the community comprise 85%-95% of total human antibacterial consumption. J Clin Pharm Ther [Internet]. 2018 [cited 2022 Oct 25];43:59–64. Available from: https://onlinelibrary.wiley.com/doi/abs/10.1111/jcpt.12610 28833324 10.1111/jcpt.12610

[2] Fleming-Dutra KE , Hersh AL , Shapiro DJ , Bartoces M , Enns EA , File TM Jr , et al. Prevalence of inappropriate antibiotic prescriptions among us ambulatory care visits, 2010-2011. JAMA [Internet]. 2016 May 3 [cited 2022 Nov 18];315:1864–1873. Available from: 10.1001/jama.2016.4151 27139059

[3] Winders HR , Royer J , Younas M , Justo JA , Bookstaver PB , Weissman SB , et al. Temporal trends in ambulatory antibiotic prescription rates in South Carolina: impact of age, gender, and resident location. Infect Control Hosp Epidemiol [Internet]. 2020 Aug [cited 2022 Nov 7];41:879–882. Available from: https://www.cambridge.org/core/journals/infection-control-and-hospital-epidemiology/article/temporal-trends-in-ambulatory-antibiotic-prescription-rates-in-south-carolina-impact-of-age-gender-and-resident-location/35832C99ADC3330F1B76E80E46B99596 32498729 10.1017/ice.2020.70

[4] Antibiotic Prescription Fill Rates Declining in the U.S. [Internet]. Blue Cross Blue Shield Blue Health Intelligence; [cited 2024 Dec 3]. Available from: https://www.bcbs.com/dA/9209b3b6ba/fileAsset/HOA-Antibiotic-Prescriptions_2017.pdf

[5] Schröder W , Sommer H , Gladstone BP , Foschi F , Hellman J , Evengard B , et al. Gender differences in antibiotic prescribing in the community: a systematic review and meta-analysis. J Antimicrob Chemother [Internet]. 2016 Jul 1 [cited 2022 Nov 7];71:1800–1806. Available from: 10.1093/jac/dkw054 27040304

[6] Barlam TF , Morgan JR , Wetzler LM , Christiansen CL , Drainoni ML . Antibiotics for respiratory tract infections: a comparison of prescribing in an outpatient setting. Infect Control Hosp Epidemiol [Internet]. 2015 Feb [cited 2022 Nov 7];36:153–159. Available from: https://www.cambridge.org/core/journals/infection-control-and-hospital-epidemiology/article/abs/antibiotics-for-respiratory-tract-infections-a-comparison-of-prescribing-in-an-outpatient-setting/54A43205696D8BCE7245AA10E47C4E29 25632997 10.1017/ice.2014.21

[7] Pinkhasov RM , Wong J , Kashanian J , Lee M , Samadi DB , Pinkhasov MM , et al. Are men shortchanged on health? Perspective on health care utilization and health risk behavior in men and women in the United States. Int J Clin Pract 2010;64:475–487. 20456194 10.1111/j.1742-1241.2009.02290.x

[8] Urban & Rural Population 2010 | South Carolina Revenue and Fiscal Affairs Office [Internet]. [cited 2024 Nov 12]. Available from: https://rfa.sc.gov/data-research/population-demographics/census-state-data-center/urban-and-rural-population-2010

[9] Kufel WD , Mastro KA , Mogle BT , Williams KS , Jester J , Snyder J , et al. Providers’ knowledge and perceptions regarding antibiotic stewardship and antibiotic prescribing in rural primary care clinics. JACCP J Am Coll Clin Pharm [Internet]. 2020 [cited 2022 Nov 8];3:601–608. Available from: https://onlinelibrary.wiley.com/doi/abs/10.1002/jac5.1198

[10] Bizune D , Tsay S , Palms D , King L , Bartoces M , Link-Gelles R , et al. Regional variation in outpatient antibiotic prescribing for acute respiratory tract infections in a commercially insured population, United States, 2017. Open Forum Infect Dis [Internet]. 2023 Feb 1 [cited 2023 Feb 21];10:ofac584. Available from: 10.1093/ofid/ofac584 PMC9905267 36776774

[11] Shi Z , Barnett ML , Jena AB , Ray KN , Fox KP , Mehrotra A . Association of a clinician’s antibiotic-prescribing rate with patients’ future likelihood of seeking care and receipt of antibiotics. Clin Infect Dis [Internet]. 2021 Oct 1 [cited 2022 Nov 7];73:e1672–9. Available from: 10.1093/cid/ciaa1173 32777032 PMC8492129

[12] Martinez KA , Rood M , Rothberg MB . Coding bias in respiratory tract infections may obscure inappropriate antibiotic use. J Gen Intern Med 2019;34:806–808. 30652274 10.1007/s11606-018-4823-x PMC6544729

