# Supplementary material for: Appropriateness of ambulatory antibiotic prescribing in South Carolina, 2012–2017
Source: Antimicrob Steward Healthc Epidemiol. 2025 Jan 15;5(1):e25. doi: 10.1017/ash.2024.500 (PMC11795426; doi:10.1017/ash.2024.500)
Supplement: Bailey et al. supplementary material [file S2732494X2400500Xsup001.docx]

Supplemental Data.

Table 1. Not Indicated Conditions and Relevant ICD codes

| **Not Indicated Condition** | **ICD9 Code** | **ICD9 Code Description** | **ICD10 Code** | **ICD10 Code Description** |
| --- | --- | --- | --- | --- |
| ****indicates all codes beginning with parent code were captured*** | | | | |
|  |  |  |  |  |
| ***Allergy*** | 477.0 | Allergic rhinitis due to pollen | J30.1 | Allergic rhinitis due to pollen |
|  | 477.1 | Allergic rhinitis due to food | J30.2 | Other seasonal allergic rhinitis |
|  | 477.2 | Allergic rhinitis due to animal (cat/dog) hair and dander | J30.5 | Allergic rhinitis due to food |
|  |  |  | J30.81 | Allergic rhinitis due to animal (cat/dog) hair and dander |
|  | 477.8 | Allergic rhinitis due to other allergen | J30.89 | Other allergic rhinitis |
|  | 477.9 | Allergic rhinitis cause unspecified | J30.9 | Allergic rhinitis cause unspecified |
|  |  |  |  |  |
| ***Asthma*** | 493* | Asthma | J45* | Asthma |
|  |  |  |  |  |
| ***Bronchitis*** | 466.0 | Acute bronchitis | J20.3 | Acute bronchitis due to coxsackievirus |
|  |  |  | J20.4 | Acute bronchitis due to parainfluenza virus |
|  |  |  | J20.5 | Acute bronchitis due to respiratory syncytial virus |
|  |  |  | J20.6 | Acute bronchitis due to rhinovirus |
|  |  |  | J20.7 | Acute bronchitis due to echovirus |
|  |  |  | J20.8 | Acute bronchitis due to other specified organisms |
|  |  |  | J20.9 | Acute bronchitis, unspecified |
|  |  |  | J21.0 | Acute bronchiolitis due to respiratory syncytial virus |
|  |  |  | J21.1 | Acute bronchiolitis due to human metapneumovirus |
|  |  |  | J21.8 | Acute bronchiolitis due to other specified organisms |
|  |  |  |  |  |
| ***COPD*** | 491.0 | Simple chronic bronchitis | J41.0 | Simple chronic bronchitis |
|  | 491.1 | Mucopurulent chronic bronchitis | J41.1 | Mucopurulent chronic bronchitis |
|  | 491.20 | Obstructive chronic bronchitis without exacerbation | J41.8 | Mixed simple and mucopurulent chronic bronchitis |
|  | 491.21 | Obstructive chronic bronchitis with exacerbation | J42 | Unspecified chronic bronchitis |
|  | 491.2 | Obstructive chronic bronchitis with acute bronchitis | J43* | Emphysema |
|  | 491.8 | Other chronic bronchitis | J44.1 | Chronic obstructive pulmonary disease with exacerbation |
|  | 491.9 | Unspecified chronic bronchitis | J44.9 | Chronic obstructive pulmonary disease unspecified |
|  | 492* | Emphysema | J47.1 | Bronchiectasis with exacerbation |
|  | 494* | Bronchiectasis | J47.9 | Bronchiectasis uncomplicated |
|  | 496 | Chronic airway obstruction not elsewhere classified |  |  |
|  |  |  |  |  |
| ***Influenza & Viral Pneumonia*** | 480* | Viral pneumonia | J10* | Influenza due to certain identified influenza viruses |
|  | 487* | Influenza caused by unspecified influenza virus, excludes pneumonia with bacterial causes | J11* | Influenza caused by unspecified influenza virus, excludes pneumonia with bacterial causes |
|  | 488* | Influenza due to certain identified influenza viruses | J12* | Viral pneumonia |
|  |  |  |  |  |
| ***Serous Otitis*** | 381* | Nonsuppurative otitis media and Eustachian tube disorders | H65* | Nonsuppurative otitis media and Eustachian tube disorders |
|  |  |  |  |  |
| ***Viral URI*** | 460 | Acute nasopharyngitis (common cold) | J00 | Acute nasopharyngitis [common cold] |
|  | 462.0 | Acute laryngopharyngitis | J06.0 | Acute laryngopharyngitis |
|  | 465.0 | Acute upper respiratory infection of multiple or unspecified sites | J06.9 | Acute upper respiratory infection, unspecified |

Table 2. Maybe Indicated Conditions and Relevant ICD codes.

|  |  |  |  |  |
| --- | --- | --- | --- | --- |
| **Maybe Indicated Condition** | **ICD9 Code** | **ICD9 Code Description** | **ICD10 Code** | **ICD10 Code Description** |
| ****indicates all codes beginning with parent code were captured*** | | | | |
|  |  |  |  |  |
| Acne | 706.0 | Acne varioliformis | L70* | Acne |
|  | 706.1 | Other acne |  |  |
|  |  |  |  |  |
| Strep Sore Throat | 034.0 | Streptococcal sore throat | J02.0 | Streptococcal pharyngitits |
|  |  |  | J02.8 | Acute pharyngitits due to other specified organisms |
|  |  |  | J03.0 | Streptococcal tonsilitis |
|  |  |  | J03.8 | Acute tonsillitis due to other specified organisms |
|  |  |  |  |  |
| Suppurative Otitis | 382* | Suppurative and unspecified otitis media | H66* | Suppurative and unspecified otitis media |
|  |  |  |  |  |
| Cellulitis | 681* | Cellulitis and abscess of finger and toe | L03* | Cellulitis and acute lymphangitis |
|  | 682* | Other cellulities and abscess (face, neck, trunk, upper arm and forearm, hand except fingersand thumb, buttock, leg except foot, foot except toes, other specified site, unspecified site) |  |  |
|  | 683 | Acute lymphangitis |  |  |
|  |  |  |  |  |
| Peritonsillar Abscess | 475* | Peritonsillar Abscess | J36* | Peritonsillar Abscess |
|  |  |  |  |  |
| Bacterial pneumonia | 481 | Pneumococcal pneumonia | J13 | Pneumonia due to Streptococcal pneumonia |
|  | 482* | Other bacterial pneumonia | J14 | Pneumonia due to Hemophilus influenzae |
|  | 483* | Pneumonia due to other specified organisms | J15* | Bacterial pneumonia not classified elsewhere |
|  | 485* | Bronchopneumonia, organism unspecified | J16* | Pneumonia in infectious diseases not elsewhere classified |
|  | 486* | Pneumonia organism unspecified | J18* | Pneumonia organism unspecified |
|  |  |  |  |  |
|  |  |  |  |  |
| Sinusitis | 461* | Acute sinusitis | J01* | Acute sinusitis |
|  |  |  |  |  |
| UTI | 590* | Infection of kidney | N10 | Acute pyelonephritis |
|  | 595* | Cystitis | N12 | Tubulo-interstitial nephritis |
|  |  |  | N151 | Renal and perinephritic abscess |
|  |  |  | N159 | Renal Tubulo-interstitial disease, unspecified |
|  |  |  | N16 | Renal Tubulo-interstitial disorders in diseases classified elsewhere |
|  |  |  | N2884 | Pyelitis cystica |
|  |  |  | N2885 | Pyeloureteritis cystica |
|  |  |  | N2886 | Ureteritis cystica |
|  |  |  | N300* | Acute cystitis |
|  |  |  | N309* | Cystitis unspecified |
|  |  |  | N390 | Urinary tract infection site not specified |
|  |  |  |  |  |
